# The structural effect of high intensity ultrasound on peritoneal tissue: a potential vehicle for targeting peritoneal metastases

**DOI:** 10.1186/s12885-020-06981-4

**Published:** 2020-05-27

**Authors:** Agata Mikolajczyk, Tanja Khosrawipour, Joanna Kulas, Pawel Migdal, Mohamed Arafkas, Jakub Nicpon, Veria Khosrawipour

**Affiliations:** 1 Department of Biochemistry and Molecular Biology, Faculty of Veterinary Medicine, Wroclaw University of Environmental and Life Sciences, 50-375 Wroclaw, Poland; 2 Division of Colorectal Surgery, Department of Surgery, University of California Irvine, California, 92868 USA; 3 Department of Surgery (A), University-Hospital Düsseldorf, Heinrich-Heine University Düsseldorf, 40225 Düsseldorf, Germany; 4 Department of Environment, Hygiene and Animal Welfare, University of Environmental and Life Sciences, 51-631 Wroclaw, Poland; 5 Department of Plastic Surgery, Ortho-Klinik Dortmund, 44263 Dortmund, Germany; 6 The Center of Experimental Diagnostics and Innovative Biomedical Technology, Wroclaw University of Environmental and Life Sciences, 50-375 Wroclaw, Poland

**Keywords:** Ultrasound, Drug penetration, Peritoneal metastasis, Chemotherapy, Peritoneum

## Abstract

**Background:**

High-intensity ultrasound (HIUS) has been increasingly investigated as a possible tool in the treatment of multiple tumor entities. However, there is only little knowledge on the effect of HIUS on the peritoneum. This preliminary study aims to investigate HIUS’ potential for altering the peritoneal surface and potentially improving current treatments for peritoneal metastases. For this purpose, HIUS’ qualitative and quantitative structural effects on the peritoneal tissue were analyzed by means of light, fluorescence and electron microscopy.

**Methods:**

Proportional sections were cut from the fresh postmortem swine peritoneum. Peritoneal surfaces were covered with a 6 mm thick liquid film of 0.9% NaCl. HIUS was applied in all tissue samples for 0 (control), 30, 60, 120 and 300 s. Peritoneal tissues were analyzed using light-, fluorescence and electron microscopy to detect possible structural changes within the tissues.

**Results:**

Following HIUS, a superficial disruption of peritoneal tissue was visible in light microscopy, which amplified with increased time of HIUS’ application. Fluorescence microscopy showed both peritoneal and subperitoneal disruption with tissue gaps. Electron microscopy revealed structural filamentation of the peritoneal surface.

**Conclusion:**

Our data indicate that HIUS causes a wide range of effects on the peritoneal tissue, including the formation of small ruptures in both peritoneal and subperitoneal tissues. However, according to our findings, these disruptions are limited to a microscopical level. Further studies are required to evaluate whether HIUS application can benefit current therapeutic regimens on peritoneal metastases and possibly enhance the efficacy of intraperitoneal chemotherapy.

## Background

High intensity ultrasound (HIUS) has been increasingly investigated as a possible tool in the treatment of many different tumor entities, e.g. cancers of prostate, kidney, liver, pancreaticobiliary and other intrabdominal malignancies [1–3]. While HIUS is still under clinical evaluation, previous studies indicate its potential to improve overall antitumoral activity regardless of chemotherapeutic applications [4]. Despite encouraging first clinical results [5], no studies have been conducted yet to assess HIUS’ possible application in the treatment of peritoneal metastases (PM). In all previous clinical applications, the HIUS beam was “focused” on a single spot in the body (High intensity focused ultrasound, HIFU). However, while PM usually covers a large surface, its depth is only minimal. This might be one reason as to why HIFU has not been considered for peritoneal applications. This study aims to modify the conventional HIFU to a “non-focused” HIUS approach and assess its potential in PM treatment. While HIUS is assumed to impact the peritoneum when used in the treatment of liver cancer, the validity of this assumption remains unclear [6]. Considering that any interaction with the peritoneum may be used in a therapeutic capacity, and inaccessibility remains one of the main difficulties in PM treatment, it seems astonishing that HIUS has never been investigated as a potential tool in PM treatment. PM is a common manifestation of advanced gastrointestinal and gynecological cancers, and affected patients usually have a very poor prognosis with median survival rates of only a few months [7]. Recent studies indicate that the combination of HIUS with intraperitoneal chemotherapy (IPC) could significantly increase drug penetration depths and therefore enhance the overall antitumoral effect, especially when applied with liposomal doxorubicin [8, 9]. While this effect has mostly been attributed to the rupture of liposomal doxorubicin [10], the results partially exceeded penetration levels observed in conventional chemotherapeutic solutions [8]. At the same time, no structural damage to the peritoneum was detected. Still, some authors have suggested that HIUS might affect the peritoneal surface when accidently applied during hepatocellular carcinoma treatment (HCC) [6]. To our knowledge, neither the application of HIUS and its potential, nor its possible side effects on the peritoneum have ever been systematically studied in the context of PM. In general surgery, HIUS is an established procedure predominantly used in the treatment of HCC [11, 12]. In a previous study, HIUS was assumed to cause local heat on the peritoneum, which could possibly induce peritoneal tissue destruction [6]. However, recent clinical evaluations indicate that HIUS might be safe for intraperitoneal use [13]. Knowing the antitumoral properties demonstrated by HIUS in HCC, it seems reasonable to assume similar effects in PM. Thus, with respect to its low invasiveness and absence of radiation, HIUS may potentially play an important role in future PM treatment. To evaluate the structural effects of HIUS on the peritoneum, we studied a well-established ex-vivo model in which we investigated peritoneal samples following HIUS application using light, fluorescence and electron microscopy.

## Methods

**Fig. 1:**
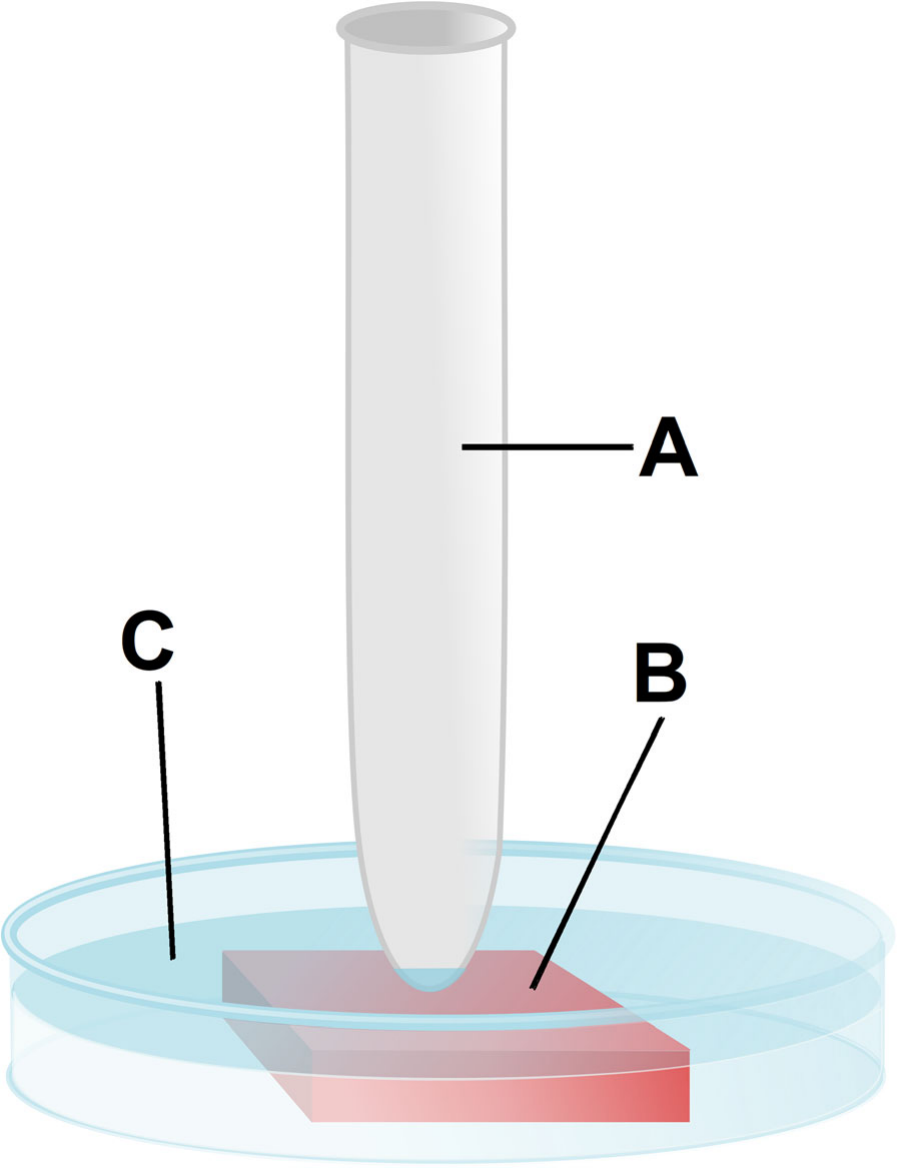
HIUS ex-vivo experiment on fresh swine peritoneum in 0.9% NaCl. The fresh full thick swine peritoneum sample is placed in a petri-dish filled with NaCl 0.9%. **a** Sonificator at 3 mm distance to the peritoneal sample. **b** Peritoneal sample. **c** NaCl 0.9%

No approval of the local board on animal welfare was required as the experiments were performed using commercially available tissue samples. A local animal supplier (Zerniki Wielkie, 55–020 Wroclaw, Poland) provided the fresh post-mortem swine peritoneum. This peritoneum was cut into proportional samples and placed into petri dishes. Then, the samples were covered with NaCl 0.9% until a layer of 6 mm covering the samples was attained (Fig. 1). HIUS (Sonopuls HD 2070, Bandelin, Berlin, Germany) at 70 W and 20 kHz was applied on the peritoneal tissue using a metal pen. The applied HIUS beam was not focused with high intensity, but rather spread from the tip of the metal pin to the periphery with continuously decreasing intensity. The tip of the pen was as close as 3 mm to the tissue. Each sample group included 3 peritoneal tissue samples and received either 30, 60, 120 or 300 s of HIUS treatment. The control group did not receive any HIUS exposure and was only placed in a petri dish for 300 s and covered by NaCl 0.9%. One sample of each group was subject to further analyses by means of light, fluorescence, or electron microscopy. Experiments were independently performed three times.

### Light microscopy (LM) analysis

Peritoneal tissue was surgically removed and placed under a light microscope (Nikon Instruments Europe B.V. Amsterdam, Netherlands) to detect major structural changes. For samples that were later used in light microcopy, a temperature probe was placed at a 3 mm distance to the tip off the metal pen to measure a possible temperature increase.

### Fluorescence microscopy (FM) analysis

The second group of samples was immediately frozen in liquid nitrogen to enable cryo sectioning (10 μm) of different areas of each specimen. To stain nuclei, sections were mounted with VectaShield containing 1.5 μg/ml 4′,6-diamidino-2-phenylindole (DAPI). Probes were analyzed using Nikon Eclipse 80i fluorescence microscope (Nikon Instruments Europe B.V. Amsterdam, Netherlands) and subperitoneal structural tissue damage was measured.

### Electron microscopy (EM) analysis

A representative amount of the tissue sample was visualized using cryogenic scanning electron microscopy (cryo-SEM).

For this purpose, tissue samples were fixed in 2.5% glutaraldehyde solution in phosphate buffer (pH = 7.2) overnight. Following fixation, samples were cleaned in phosphate buffer, rinsed in ultrapure (filtered through 0.1um syringe filter) deionized water, mounted on cryo shuttle using OCT/colloidgraphite mixture and plunged in liquid nitrogen. Then, frozen samples were quickly transferred to the cryo-preparation chamber (Cryo Quorum PP3010T) and sputtered with a conductive platinum layer at -140C. In the next step, samples were transferred to the microscope chamber maintaining the same temperature of -140C (Auriga60, Zeiss) and observed at 2 kV of acceleration voltage using In Lens and SE2 secondary electron detectors.

### Statistical analyses

Experiments were independently performed three times. Sigma Plot 12 (Systat Software Inc., California, USA) was used to perform statistical analysis. For analyses of independent groups, the Kruskal-Wallis One Way Analysis of Variance on Ranks was utilized. A *p*-value of < 0.05 was considered significant.

## Results

### Light microscopy (LM)

When probes were removed for further analysis, macroscopic changes on the peritoneal surface became detectable. Macroscopically, the peritoneum had become more whitish and presumably thicker. No visual signs of tissue tearing were detectable. There was no perforation in the peritoneal layer. The peritoneal surface became jelly-like after medium was removed for further preparation. Using a temperature probe, no temperature increase was detectable in the medium neither during the experiment and nor immediately after. After removal of the peritoneum for further light microscopy, the clear structural texture of the tissue was more whitish when compared to untreated samples. No clear signs of larger tissue disruptions were visible.

### Fluorescence microscopy (FM)

**Fig. 2:**
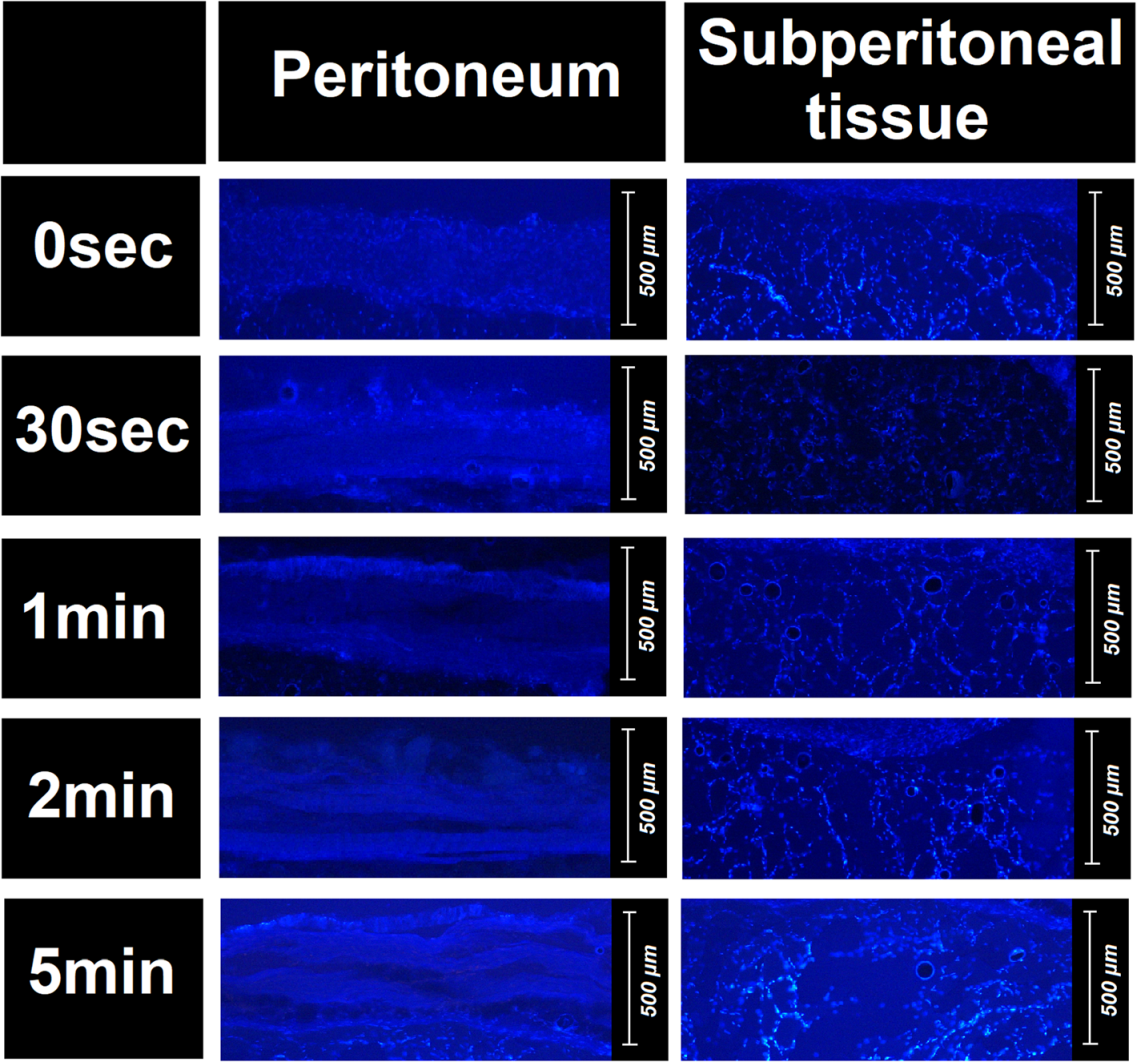
Tissue disruption following HIUS at different durations. Left side: changes on the peritoneum. Right side: changes of the subperitoneal tissue

**Fig. 3:**
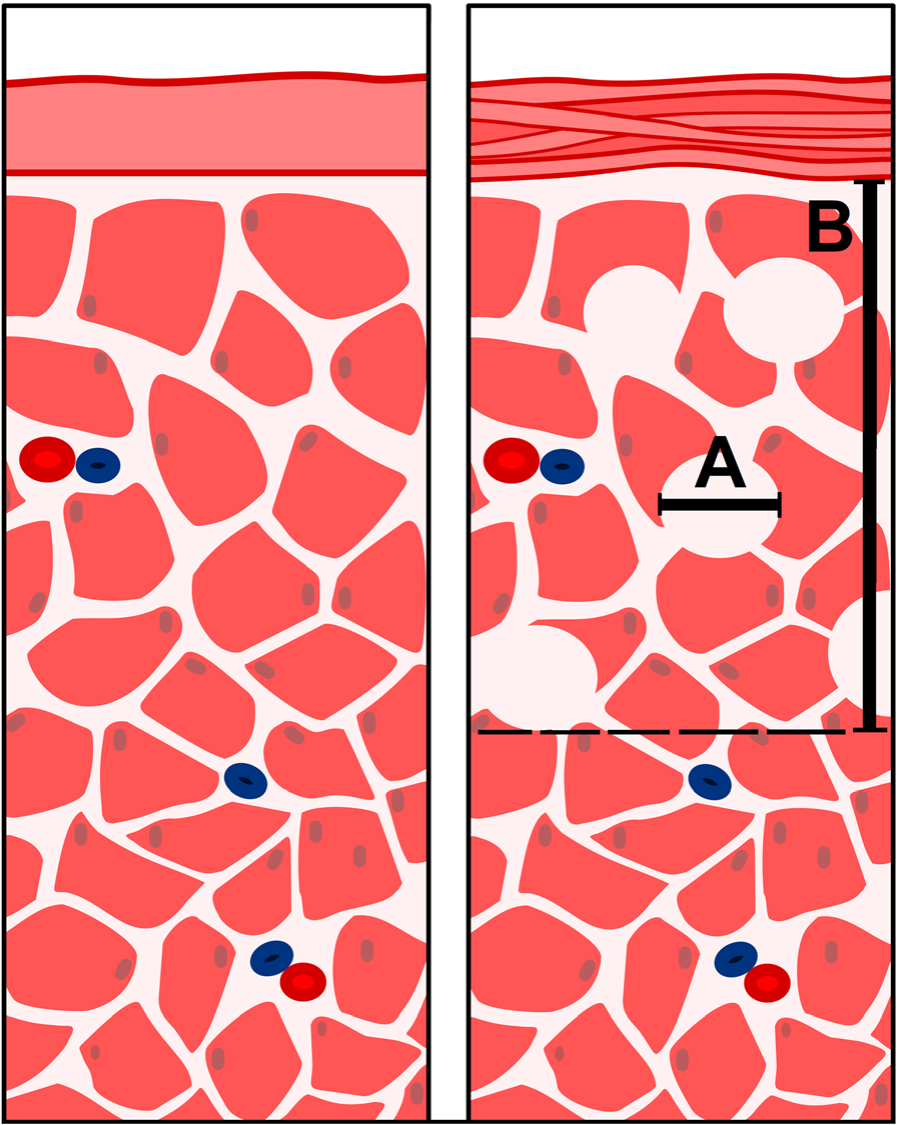
Microscopic model of HIUS’ effects on the peritoneal tissue. Left side: untreated tissue. Right side: HIUS treated peritoneum. **a** horizontal disruptions. **b** total disruption depth into subperitoneal tissue

**Fig. 4:**
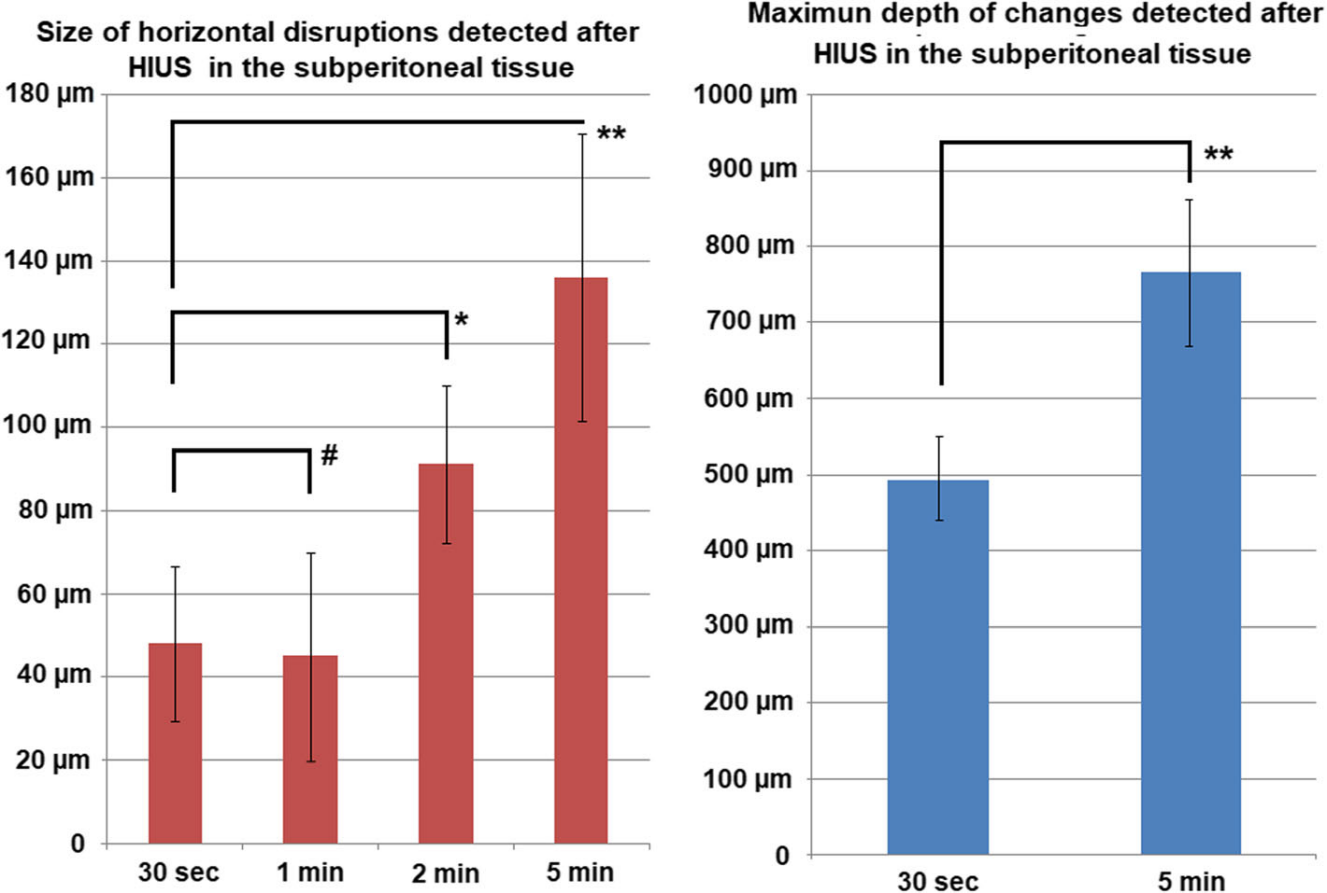
Left side: Size of horizontal disruptions following HIUS. Right side: Disruption depth into the subperitoneal tissue following HIUS. # = *p* > 0.05, * = *p* < 0.05, ** = *p* < 0.01

Microscopic analysis of tissue samples showed a substantial structural difference compared to the control group. The superficial peritoneal layer of the samples showed signs of structural mechanical disintegration with ongoing HIUS duration (Figs. 2 and 3). The superficial peritoneal layer seemed to be disrupted into horizontal fibers. This effect seemed to increase with continuous treatment (Figs. 2 and 3). While in tissue samples with short HIUS exposure time, this structural disintegration was limited to some areas of the peritoneum, in probes treated for 120 s and longer these disintegrated areas fused and created several parallel lines of peritoneal filaments. Additionally, the subperitoneal muscle tissue was disrupted. However, disruptions were rather vertical than horizontal. Also, vertical disruption was observed to increase with longer exposure time to HIUS (Figs. 2 and 3). This increase in disruption size was significant from 48 +/− 18,5 μm to 153 +/− 34,5 μm (*p* < 0.01) (Fig. 4). Disruption depth into the subperitoneal tissue was measured (Fig. 4) and increased significantly from 494 +/− 54,1 μm to 765 +/− 96,7 μm (*p* < 0.01).

### Electron microscopy (EM)

**Fig. 5:**
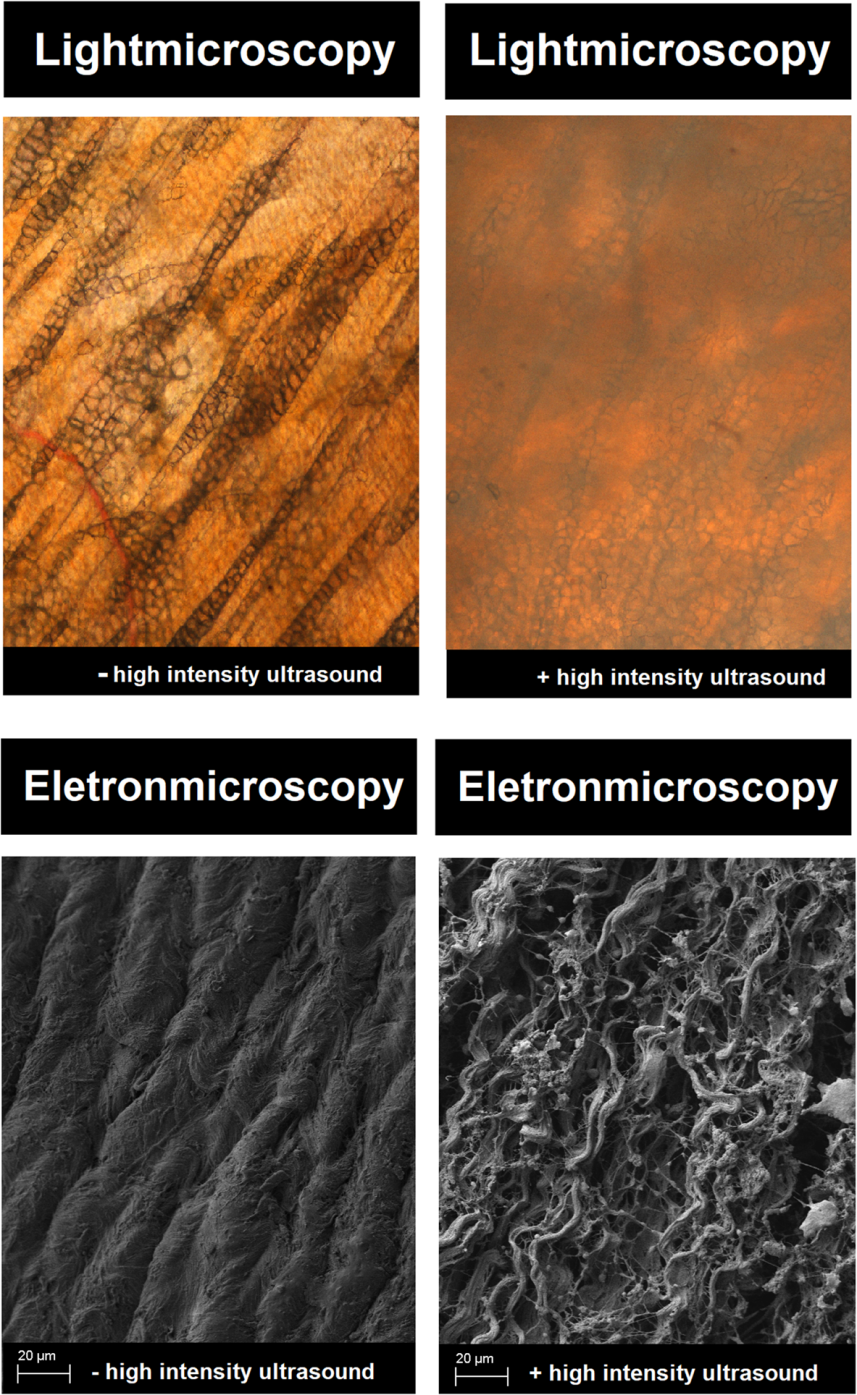
Light microscopy and Cryogenic scanning electron microscopy of peritoneal surface with (+) and without (−) HIUS, magnification level 1000X

The applied magnification was at a wide range between 500X and 5000X. Structural disintegration of the uppermost peritoneal layer was confirmed by EM in probes treated with (+) versus probes without (−) HIUS. The peritoneal surface was practically divided into bundles of fibers (Fig. 5). In contrast, untreated probes showed a compact and mostly smooth surface.

## Discussion

While in the past few years, many improvements have been observed in chemotherapeutic regimens and new drug compositions, a significant amount of PM patients fail to respond to systemic and local treatments. This circumstance is mostly attributed to molecular mechanisms and limited drug distribution into the tumor [14]. Similar limitations have been observed in IPC during PM treatments [15, 16]. However, higher local drug disposition and increased tissue drug penetration is reported to enhance the anti-tumoral effect [17–19]. Attempts to improve tissue penetration rates by treating the peritoneal surface prior to chemotherapeutic application were mostly unsuccessful. For example, concepts like using an energy beam via radiation to prepare the peritoneal tissue for IPC have unfortunately not shown any improved penetration effects [20–22]. However, our data suggests that HIUS might be an easy, feasible additional feature in the treatment of PM. While our data is limited, and the study is preliminary in nature, our findings present the potential effects of HIUS on the peritoneum. In the future, these effects can be used in various applications. By creating very small tissue disruptions within the peritoneal surface, the transport of various particles through this main barrier is facilitated, resulting in significantly improved penetration rates of chemotherapeutic drugs. Some previous studies suggest these possible HIUS effects in combination with chemotherapy [8, 23]. However, these studies were primarily investigating drug tissue penetrations without emphasizing structural tissue changes, thus giving little explanation for this effect. A very recent study has, for the first time, analyzed drug penetration on the peritoneum following HIUS application, and the findings of this study indicate that penetration rates can be enhanced by more than threefold depending on the duration of the HIUS beam [24]. Since the effect of HIUS seems to show limitations in depth, it could be used for PM treatment during cytoreductive surgery to possibly disrupt the vascular network of single nodules. This concept is quite interesting since tumor nodules in PM are assumed to have a reduced blood supply compared to regular peritoneal tissue [25]. Other HIUS aspects. e.g. its role in the enhanced apoptosis of cancer cells has been recently discovered and requires further analysis [26]. Thus, HIUS potential for PM must be further investigated and warrants more studies to thoroughly investigate its potential. However, this present study offers important first insight of potential HIUS application to treat PM.

## Conclusions

Our data indicate that HIUS creates disruptions in the peritoneal surface and its underlying tissues. In the subperitoneal tissue, HIUS application results in microbubble formation. Beside its direct effects on the peritoneum, these structural surface changes might also result in increased drug permeability.

To adequately assess HIUS’ efficacy as well as its therapeutic possibilities on the peritoneum, further studies are required.

## Data Availability

The datasets used and/or analysed during the current study are available from the corresponding author on reasonable request.

## Abbreviations

C02: Carbon dioxide
CRS: Cytoreductive surgery
EM: Electron microscopy
LM: Light microscopy
FM: Fluorescence microscopy
HCC: Hepatocellular carcinoma
HIUS: High intensity ultrasound
HIPEC: Hyperthermic intra-peritoneal chemotherapy
IAP: Intra-abdominal pressure
IPC: Intra-peritoneal chemotherapy
PM: Peritoneal metastasis

## References

[1] Zhao J, Zhao F, Shi Y, Deng Y, Hu X, Shen H (2017). The efficacy of a new high intensity focused ultrasound therapy for locally advanced pancreatic cancer. J Cancer Res Clin Oncol.

[2] Siu JY, Liu C, Zhou Y (2017). High-intensity focused ultrasound ablation around the tubing. PLoS One.

[3] Maloney E, Hwang JH. Int J Hyperthermia. 2015; 31(3):302–309. 10.3109/02656736.2014.969789 25367011

[4] Feng G, Hao L, Xu C, Ran H, Zheng Y, Li P, Cao Y, Wang Q, Xia J, Wang Z. Int J Nanomedicine. 2017; 28(12):4647–4659. 10.2147/IJN.S135391 PMC5500488 28721041

[5] Xiaoping L, Leizhen Z (2013). Advances of high intensity focused ultrasound (HIFU) for pancreatic cancer. Int J Hyperth.

[6] Wu CC, Chen WS, Ho MC, Huang KW, Chen CN, Yen JY, Lee PH (2008). Minimizing abdominal wall damage during high-intensity focused ultrasound ablation by inducing artificial ascites. J Acoust Soc Am.

[7] Facy O, Al Samman S, Magnin G, Ghiringhelli F, Ladoire S, Chauffert B, Rat P, Ortega-Deballon P (2012). High pressure enhances the effect of hyperthermia in intraperitoneal chemotherapy with oxaliplatin: an experimental study. Ann Surg.

[8] Mikolajczyk A, Khosrawipour V, Kulas J, Kocielek K, Migdal P, Arafkas M, Khosrawipour T. Release of doxorubicin from its liposomal coating via high intensity ultrasound. Mol Clin Oncol. 2019;11(5):483–7. 10.3892/mco.2019.1917 PMC6787992 31620279

[9] Mikolajczyk A, Khosrawipour V, Schubert J, Grzesiak J, Chaudhry H, Pigazzi A, Khosrawipour T (2018). Effect of Liposomal Doxorubicin in Pressurized Intra-Peritoneal Aerosol Chemotherapy (PIPAC). J Cancer.

[10] Santos Marc A., Goertz David E., Hynynen Kullervo (2017). Focused Ultrasound Hyperthermia Mediated Drug Delivery Using Thermosensitive Liposomes and Visualized With in vivo Two-Photon Microscopy. Theranostics.

[11] Huang L, Zhou K, Zhang J, Ma Y, Yang W, Ran L, Jin C, Dimitrov DD, Zhu H (2019). Efficacy and safety of high-intensity focused ultrasound ablation for hepatocellular carcinoma by changing the acoustic environment: microbubble contrast agent (SonoVue) and transcatheter arterial chemoembolization. Int J Hyperth.

[12] Daecher A, Stanczak M, Liu JB, Zhang J, Du S, Forsberg F, Leeper DB, Eisenbrey JR (2017). Localized microbubble cavitation-based antivascular therapy for improving HCC treatment response to radiotherapy. Cancer Lett.

[13] Strunk HM, Lützow C, Henseler J, Mücke M, Rauch M, Marx C, Schild HH, Marinova M (2018). Mesenteric vessel patency following HIFU therapy in patients with locally invasive pancreatic Cancer. Ultraschall Med.

[14] Jain RK (1994). Barriers to drug delivery in solid tumors. Sci Am.

[15] Khosrawipour V, Mikolajczyk A, Schubert J, Khosrawipour T (2018). Pressurized Intra-peritoneal Aerosol Chemotherapy (PIPAC) via Endoscopical Microcatheter System. Anticancer Res.

[16] Khosrawipour T, Wu D, Bellendorf A, Mohanaraja KE, Diaz-Carballo D, Khosrawipour V (2017). Feasibility of Single Tumorspot treatment in Peritoneal Carcinomatosisi via Close range Doxorubicin impaction in Pressurized Intra-Peritoneal Aerosol Chemotherapy (PIPAC). J Clin Exp Oncol.

[17] Khosrawipour V, Khosrawipour T, Falkenstein TA, Diaz-Carballo D, Förster E, Osma A, Adamietz IA, Zieren J, Fakhrian K (2016). Evaluating the Effect of Micropump© Position, Internal Pressure and Doxorubicin Dosage on Efficacy of Pressurized Intra-peritoneal Aerosol Chemotherapy (PIPAC) in an Ex Vivo Model. Anticancer Res.

[18] Khosrawipour V, Diaz-Carballo D, Acikelli AH, Khosrawipour T, Falkenstein TA, Wu D, Zieren J, Giger-Pabst U (2016). Cytotoxic effect of different treatment parameters in pressurized intraperitoneal aerosol chemotherapy (PIPAC) on in vitro proliferation of human colonic cancer cells. World J Surg Oncol..

[19] Schubert J, Khosrawipour V, Chaudhry H, Arafkas M, Knoefel WT, Pigazzi A, Khosrawipour T (2019). Comparing the cytotoxicity of taurolidine, mitomycin C, and oxaliplatin on the proliferation of in vitro colon carcinoma cells following pressurized intra-peritoneal aerosol chemotherapy (PIPAC). World J Surg Oncol.

[20] Khosrawipour V, Giger-Pabst U, Khosrawipour T, Pour YH, Diaz-Carballo D, Förster E, Böse-Ribeiro H, Adamietz IA, Zieren J, Fakhrian K (2016). Effect of Irradiation on Tissue Penetration Depth of Doxorubicin after Pressurized Intra-Peritoneal Aerosol Chemotherapy (PIPAC) in a Novel Ex-Vivo Model. J Cancer.

[21] Khosrawipour V, Khosrawipour T, Hedayat-Pour Y, Diaz-Carballo D, Bellendorf A, Böse-Ribeiro H, Mücke R, Mohanaraja N, Adamietz IA, Fakhrian K (2017). Effect of Whole-abdominal Irradiation on Penetration Depth of Doxorubicin in Normal Tissue After Pressurized Intraperitoneal Aerosol Chemotherapy (PIPAC) in a Post-mortem Swine Model. Anticancer Res.

[22] Khosrawipour V, Bellendorf A, Khosrawipour C, Hedayat-Pour Y, Diaz-Carballo D, Förster E, Mücke R, Kabakci B, Adamietz IA, Fakhrian K (2016). Irradiation Does Not Increase the Penetration Depth of Doxorubicin in Normal Tissue After Pressurized Intra-peritoneal Aerosol Chemotherapy (PIPAC) in an Ex Vivo Model. In Vivo.

[23] Lyon PC, Griffins LF, Lee J, Chung D, Carlise R, Wu F, Middelton MR, Gleeson FV, Coussios CC (2017). Clinical trial protocol for Tardox: a phase I study to investigate the feasibility of target release of lyso-thermosensitive liposomal doxorubicin (ThermoDox) using focused ultrasound in patients with liver tumors. J Ther Ultrasound.

[24] Khosrawipour V, Reinhard S, Martino A, Khosrawipour T, Arafkas M, Mikolajczyk A (2019). Increased Tissue Penetration of Doxorubicin in Pressurized Intraperitoneal Aerosol Chemotherapy (PIPAC) after High-Intensity Ultrasound (HIUS). Int J Surg Oncol.

[25] Kastelein Arnoud W., Vos Laura M. C., van Baal Juliette O. A. M., Koning Jasper J., Hira Vashendriya V. V., Nieuwland Rienk, van Driel Willemien J., Uz Zühre, van Gulik Thomas M., van Rheenen Jacco, Ince Can, Roovers Jan-Paul W. R., van Noorden Cornelis J. F., Lok Christianne A. R. (2020). Poor perfusion of the microvasculature in peritoneal metastases of ovarian cancer. Clinical & Experimental Metastasis.

[26] Ning Z, Zhu Z, Wang H, Zhang C, Xu L, Zhuang L, Yan X, Wang D, Wang P, Meng Z (2019). High-intensity focused ultrasound enhances the effect of bufalin by inducing apoptosis in pancreatic cancer cells. Onco Targets Ther.

